# S71. ABERRANT TIMING AND SALIENCE NETWORK IN SCHIZOPHRENIA: FINDINGS FROM A META-ANALYSIS

**DOI:** 10.1093/schbul/sby018.858

**Published:** 2018-04-01

**Authors:** Irene Alústiza, María Sol Garcés, Marta Ortuño, Patricio Molero, Felipe Ortuño

**Affiliations:** Clinica Universidad de Navarra

## Abstract

**Background:**

Schizophrenia (SZ) affects several domains of cognitive function. Abnormal time and novelty processing, which is related to change detection, has been reported in this disorder. Timing and oddball tasks can be used to assess change detection in perceptual processes. We hypothesize that an impaired timing network underlies disruptive cognitive functioning in SZ, such as saliency detection. Therefore, timing dysfunction might be a primary cognitive deficit in this disorder.

To address this issue, our aim was to elucidate the neural areas underlying target detection and timing in SZ, as well as to determine whether the timing dysfunctional activity pattern showed by SZ patients matches the pattern involved in attention salience processing. The final purpose of our study was to identify the brain structures activated during both timing and oddball tasks in patients with SZ, as compared to healthy controls (HC).

**Methods:**

We conducted two independent comprehensive literature searches of whole-brain functional magnetic resonance imaging (fMRI) studies that compared patients with SZ and HC using oddball and timing tasks. The searches were conducted with PubMed engine up to November 2017. Keywords used in the first search were: “schizophrenia” plus “functional magnetic resonance imaging” or “fMRI” plus “timing” or “time perception” or “time estimation”. In the second search keywords used were: “schizophrenia” plus “functional magnetic resonance imaging” or “fMRI” plus “event-related”, plus “oddball”.

We excluded studies that 1) used a region-of-interest approach; 2) did not report peak coordinates for the relevant contrast; 3) used different statistical thresholds in different regions of the brain; 4) used techniques other than fMRI; 5) were based on Independent Component Analysis; 6) were case reports, qualitative studies, reviews or meta-analyses.

We ran two independent signed differential mapping (SDM) meta-analyses of fMRI studies conducting comparisons between HC and patients with SZ: one reporting brain activation patterns during an oddball task, and a second one using timing tasks. We carried out a final multimodal meta-analysis to combine the findings from the two previous SDM meta-analyses. The aim of this multimodal analysis was to detect brain regions that are activated or deactivated by both timing and oddball tasks in SZ.

**Results:**

Our initial search returned 173 papers, but application of inclusion criteria reduced this number to 8. Among them, 3 studied timing (which included a total of 53 SZ patients and 60 HC) and 5 examined oddball paradigm (which included a total of 100 SZ patients and 122 HC).

Relative to HC, patients with SZ showed significantly hypoactivation in right striatum, right middle frontal gyrus (BA 9 and 45), and right median cingulate / paracingulate gyri (BA 32) during timing tasks. For oddball tasks, even if they showed significantly decreased activation in right inferior parietal gyri (BA 40) and corpus callosum, they also exhibited hyperactivation or failure of deactivation in left superior frontal gyrus, and dorsolateral (BA 9). Finally, overlapping was found in regions that were hypoactivated and hyperactivated by oddball tasks in SZ patients relative to HC.

**Discussion:**

Our results show that there is a common dysfunctional participation of frontal, cingulate, striatum, and parietal regions in SZ during both timing and oddball tasks. These findings suggest that a deficient timing network underlies attentional salience. However, these results are preliminary and further studies may be conducted to address the specific role of timing on cognition.

